# Who interacts with whom? Social mixing insights from a rural population in India

**DOI:** 10.1371/journal.pone.0209039

**Published:** 2018-12-21

**Authors:** Supriya Kumar, Mudita Gosain, Hanspria Sharma, Eric Swetts, Ritvik Amarchand, Rakesh Kumar, Kathryn E. Lafond, Fatimah S. Dawood, Seema Jain, Marc-Alain Widdowson, Jonathan M. Read, Anand Krishnan

**Affiliations:** 1 Department of Behavioral and Community Health Sciences, Graduate School of Public Health, University of Pittsburgh, Pittsburgh, PA, United States of America; 2 Department of Community Medicine, All India Institute of Medical Sciences, New Delhi, India; 3 Delhi University, New Delhi, India; 4 Department of Epidemiology, Graduate School of Public Health, University of Pittsburgh, Pittsburgh, PA, United States of America; 5 Centers for Disease Control and Prevention, Atlanta, GA, United States of America; 6 Centers for Disease Control and Prevention, Nairobi, Kenya; 7 Centre for Health Informatics, Computing, and Statistics, Lancaster Medical School, University of Lancaster, Lancaster, England, United Kingdom; The University of Hong Kong, CHINA

## Abstract

Acute lower respiratory infections (ALRI) are a leading cause of morbidity and mortality globally, with most ALRI deaths occurring in children in developing countries. Computational models can be used to test the efficacy of respiratory infection prevention interventions, but require data on social mixing patterns, which are sparse in developing countries. We describe social mixing patterns among a rural community in northern India. During October 2015-February 2016, trained field workers conducted cross-sectional face-to-face standardized surveys in a convenience sample of 330 households in Faridabad District, Haryana State, India. Respondents were asked about the number, duration, and setting of social interactions during the previous 24 hours. Responses were compared by age and gender. Among the 3083 residents who were approached, 2943 (96%) participated, of whom 51% were male and the median age was 22 years (interquartile range (IQR) 9–37). Respondents reported contact (defined as having had a face-to-face conversation within 3 feet, which may or may not have included physical contact) with a median of 17 (IQR 12–25) people during the preceding 24 hours. Median total contact time per person was 36 person-hours (IQR 26–52). Female older children and adults had significantly fewer contacts than males of similar age (Kruskal-Wallis χ2 = 226.59, p<0.001), but spent a longer duration in contact with young children (Kruskal-Wallis χ2 = 27.26, p<0.001), suggesting a potentially complex pattern of differential risk of infection between genders. After controlling for household size and day of the week, respondent age was significantly associated with number and duration of contacts. These findings can be used to model the impact of interventions to reduce lower respiratory tract infections in India.

## Introduction

Acute lower respiratory infections (ALRIs) are major contributors to morbidity and mortality globally, especially in developing nations [1–5]. Globally, ALRI results in an estimated 2.7 million deaths per year [6]. Among children less than five years old, an estimated 704,000 ALRI deaths occurred in 2015, accounting for 12% of deaths in this age group [6]. The majority of ALRI deaths in children less than five years old occur in developing countries [6, 7].

Influenza vaccines have been available for over 50 years, and are increasingly used in young children. Respiratory Syncytial Virus (RSV) vaccines are in late stage development and may be available for routine use in the near future. Influenza viruses and RSV are estimated to cause 13% and 22% of ALRI cases in children 0–4 years old, respectively [8, 9]. Whereas young infants bear the highest burden of ALRI, school-aged children are thought to play a key role in driving epidemics of respiratory viruses due to their close contacts with other children at home and in schools. Thus, vaccination of school-age children [10–12] and school closure [13] have been proposed as interventions for reducing the burden of influenza and RSV illness among both children and other age groups. A second strategy, influenza and RSV vaccination of pregnant women, has been proposed to protect young infants who are not eligible to receive vaccine [14].

Computational models are a potential tool for assessing the effectiveness of interventions to reduce the burden of influenza and RSV-associated ALRI. A common approach is to model the population as compartments with distinct infection status, requiring knowledge of the rate at which susceptible individuals come into contact with infectious individuals or their ‘contact rate’ [15]. Such age-stratified models also utilize the rates of mixing between age groups in a community of interest as an input [16]. Studies have universally observed that social mixing is age-assortative—i.e. people tend to mix with others their own age—though intergenerational contacts, such as parent-child interactions, and differences by gender exist between studies [17–24]. These data are key to model disease transmission but may vary from context to context depending on cultural norms in different communities and societies. Knowledge of who-mixes-with-whom [25] is, however, currently lacking for populations in India, a country with 18% of the world’s population [26] and limited healthcare infrastructure, where sporadic reports of avian influenza occur [27].

Contact diaries that allow respondents to self-report on their daily interactions with others are a common method for capturing social mixing data from a large representative sample of individuals [28, 29]. Contact diaries record the sociodemographic information of study respondents and ask them to characterize the nature of all contacts they make throughout a given time period. For the study of respiratory illness, surveys seek to capture encounter information pertinent to the transmission of common respiratory pathogens: this includes the length and quantity of contact events, where a contact event is defined as face-to-face encounters with others that included talk and/or touch. Self-reported contacts from European populations have proved to be a good proxy for contact rate parameters in models of airborne or droplet-borne infectious disease transmission [30], though validity of self-reported contacts may vary across populations.

We conducted a social mixing study in a rural area in Ballabgarh, Haryana, 36 km southeast of New Delhi, the capital of India. The study aimed to describe the social contact patterns of individuals in sampled households in five villages. We present analyses of mixing patterns, including age-based mixing matrices. In order to examine whether males and females have different numbers or locations of contacts in Haryana, which is known to be a patriarchal society, we also examine correlates of mixing among women and men, and present hypotheses regarding infection risk in this rural Indian population.

## Materials & methods

### Field site

Our field site was in Ballabgarh, Faridabad district, Haryana, India. This is a rural, farm-owning population, in which multi-generational households are the norm. We selected a convenience sample of 330 households in five villages, representing 14% of households in an existing ARI surveillance platform that included weekly household visits by trained health workers to capture ARI and influenza episodes among children <10 years old and adults >60 years old [31, 32]. A household was defined as people who ate meals cooked in the same kitchen, as reported in the most recent census of this population, conducted in May 2016.

### Ethical review

This study was reviewed and approved by the ethics boards at the All India Institute of Medical Sciences, New Delhi (IEC/NP-121/10-4-2015), as well as at the University of Pittsburgh (PRO15100147) and the Centers for Disease Control and Prevention, Atlanta (FWA 00014191). We received written consent from all participants, with caregivers providing written consent for participants below 7y age.

### Contact diary survey

Each resident of sampled households was approached for consent at the beginning of the study; a caregiver consented for children. At each participating household, a structured questionnaire of social contacts over the past 24 hours was administered in a face-to-face interview with each respondent. All individuals in each household were interviewed; a caregiver responded for children five years old or younger, whereas children 6–10 years old responded in the presence of a caregiver. All respondents 12-18y age were interviewed by an interviewer of the same gender as that of the respondent. A contact was defined as having had a face-to-face conversation within 3 feet, which may or may not have included physical contact. Respondents reported the age and sex of contacts, along with the total duration of encounter(s), place of contact (at home, work, school, during transport, or other), and location of the contact of maximum duration (geocoded). Respondents could report an encounter with multiple individuals as a “group” contact, including the number and age range of people in the group, and average duration of contact with each individual in the group.

### Number of contacts

We first calculated the total number of individual people reported as contacts by each respondent over the previous day, and then added the total number of people in reported groups to estimate the total number of people contacted by a respondent on the previous day.

### Contact setting

Interviewers recorded the address where the group contact or longest encounter with each person (who was reported as a contact by the respondent) occurred. All addresses were located on Google maps (https://www.maps.google.com) and geocoded (the latitude and longitude recorded) by a field staff member.

### Duration of contacts

The amount of time spent with each contacted person was recorded on a categorical scale: “less than 5 minutes,” “5 to 14 minutes,” “15 to 59 minutes,” “1 to 4 hours,” and “greater than 4 hours.” We drew 1000 random samples from a uniform distribution over each response option: for example, if a person reported that she spent 5–14 minutes with a contact, we picked a duration randomly from a uniform integer interval between 5 and 14 minutes. We estimated the duration of the contact as the average over these multiple random samples. If a person reported spending >4h with a contact, we estimated the duration of contact between 241 minutes (4.016h) and 8h (the usual maximum for a working day). For each respondent, the total amount of time spent in contact was calculated as the sum of duration of all encounters reported by the respondent.

### Age-specific contact number and duration

We calculated contact numbers and contact duration by respondent age-group (six groups: <1y, 1-4y, 5-19y, 20-34y, 35-64y, > = 65y). We used descriptive statistics, including boxplots, and the Kruskal-Wallis non-parametric H test [33] to examine contact rate differences by respondent gender and age category, and Dunn’s test to examine pairwise differences with Bonferroni correction for multiple comparisons [34].

### Age-assortative mixing matrices

To facilitate the use of these data in future mathematical models of disease transmission, we calculated age-assortative mixing matrices using the number of contacts, or the duration of contacts as our variables of interest.

To examine if the number of contacts between age groups deviated from what we might expect if mixing were random, we calculated the ratio of observed number (and duration) of contacts between age category dyads to the expected number (and duration) of contacts if mixing were proportional to census population counts; we used census population counts for rural Faridabad, Haryana, from the 2011 census of India [35]. If individuals in our sample were mixing randomly, then we would expect to observe individuals’ contact to be distributed across age groups proportional to the size of the age-groups in the population. If, however, individuals are mixing assortatively by age–where individuals preferentially mix with others of similar age to themselves–we would expect to see more contact with their own age group than expected through random mixing alone, yielding a ratio greater than one. We multiplied the population proportion in each age group from the census to the number or duration of contacts reported by respondents in each age category to find the expected contacts if mixing were random. We present these observed to expected ratios along with 95% CIs from 1,000 bootstrapped samples of respondents’ contact numbers (or durations). Ratios greater than one signify greater than expected mixing between the two age-categories. Hypothesizing that age-assortative mixing matrices may differ by gender in a patriarchal society, we also present matrices stratified by respondent gender; these serve to generate hypotheses about the gender-specific risks of infection that may arise due to the number and duration of contacts with children. We aggregated the <1 year and 1–4 year categories, as well as the 20–34 year and 35–64 year categories in order to increase the sample size in each age category.

### Regression models to examine differences in contacts by gender and to identify independent correlates of contacts

We built univariate negative binomial regression models stratified by gender with number of contacts as the outcome, and age splines as predictors to examine the differences in contacts by gender. We fitted a cubic spline to age using the flexcurv command in STATA [36]s, with reference points at 0, 5, 10, 20, 35, 60, and 80 years old. These age splines were the independent variable in a model with contact number as the outcome. We predicted contact number from this model and plotted it to examine differences by gender in the smoothed age-specific contact numbers.

We also fitted multivariate negative binomial regression models to examine the independent impact of age (categorical), household size, and weekend (Saturday or Sunday versus other days) on the number and duration of contacts. Given the different pattern of contacts reported by males and females in this study, we stratified the model by gender. We used age categories <1y, 1-4y, 5-19y, 20-34y, 35-59y, and > = 60 years old rather than the splines to ease interpretation. The age category 5–19 years old (the group with the greatest number of contacts in previous studies [18, 37]) was used as the reference category. Variance estimators accounted for clustering at the household level. We present adjusted rate ratios from these regression models.

## Results

Between October 20,^,^ 2015 and February 29, 2016 (corresponding to the winter season, when a minor peak in influenza is observed annually in the region around Delhi [38]), we approached 3083 individuals in 330 households; 2943 individuals (95.5%) were available and willing to participate in the survey. Characteristics of those included in the survey are presented in Table 1. The respiratory infection status of individuals and correlation, if any, with contact numbers and duration, will be reported separately. Respondents reported contacting a total of 47,558 individuals, and 24,931 people in group-settings for a total of 72,489 total contacts.

**Table 1:** Characteristics of respondents stratified by gender.

Characteristic		n	Males n (%)	Females n (%)
**Total**		2943	1505 (51.1)	1438 (48.9)
**Age**	<1y	73	43 (58.9)	30 (41.1)
	1-4y	305	173 (56.7)	132 (43.3)
	5-19y	964	502 (52.1)	462 (47.9)
	20-34y	776	364 (46.9)	412 (53.1)
	35-59y	445	249 (56.0)	196 (44.0)
	60y+	380	174 (45.8)	206 (54.2)
**Household size**	2–6	490	262 (53.5)	228 (46.5)
	7–9	738	385 (52.2)	353 (47.8)
	10–13	876	439 (50.1)	437 (49.9)
	14+	831	414 (49.8)	417 (50.2)
**Day of the week**	Weekday	2124	1040 (49.0)	1084 (51.0)
	Weekend	819	465 (56.8)	354 (43.2)

### Number of contacts

The median number of contacts with the past 24 hours was reported as 17 per respondent (interquartile range (IQR) 12–25). The number of contacts differed by age-category (Fig A in S1 Appendix; Kruskal-Wallis χ^2^ = 199.04, dof = 5, p<0.001). Infants had the fewest contacts compared to every other age category (p<0.01), whereas 5–19 year old respondents had the highest number of contacts (p<0.001). The number of contacts was positively correlated with household size (spearman’s rho = 0.258; p<0.001).

### Duration of contacts

The median total time spent in contact was 36 person-hours (IQR 26–52); which represents a product of the total persons met in the day with the time spent with them. Person-hours is a measure of the hours for which the respondent has been exposed to others. Time spent in contact differed by age-category (Kruskal-Wallis χ^2^ = 401.34, dof = 5, p<0.001). Persons 1–4 and 5–19 years old had more person-hours in contact than any other age category (p<0.001), whereas adults > = 60 years old had the lowest duration in contact (p<0.05) (Fig B in S1 Appendix). There was an increase in the proportion of contacts that were physical as duration of contacts increased (Fig C in S1 Appendix). A greater proportion of contacts that occurred daily were reported to be physical than those that had occurred for the first time (Fig D in S1 Appendix).

### Age-assortativity in contacts

**Fig 1:**
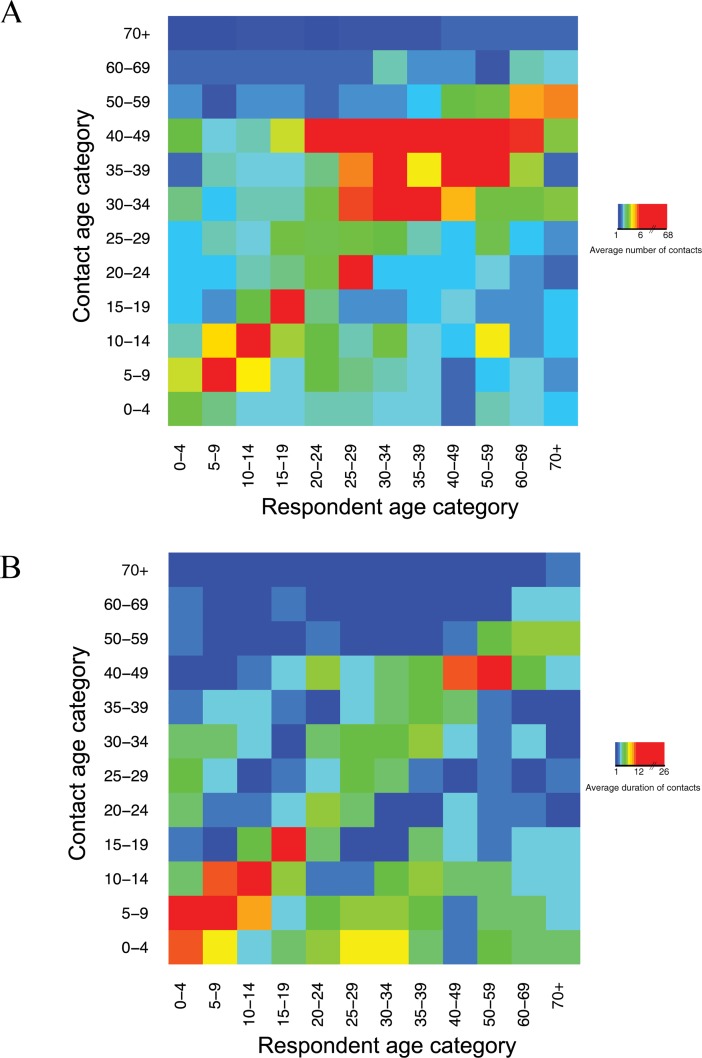
Average number of contacts reported by respondents with contacts of each age-group (A). Average number of person-hours spent by respondents in contact with people of each age-group (B).

Age-assortativity was observed in the number (Fig 1A) and duration (Fig 1B) of contacts reported by respondents. Brighter colors outside the diagonal are also apparent especially in duration of contacts (Fig 1B).

**Fig 2:**
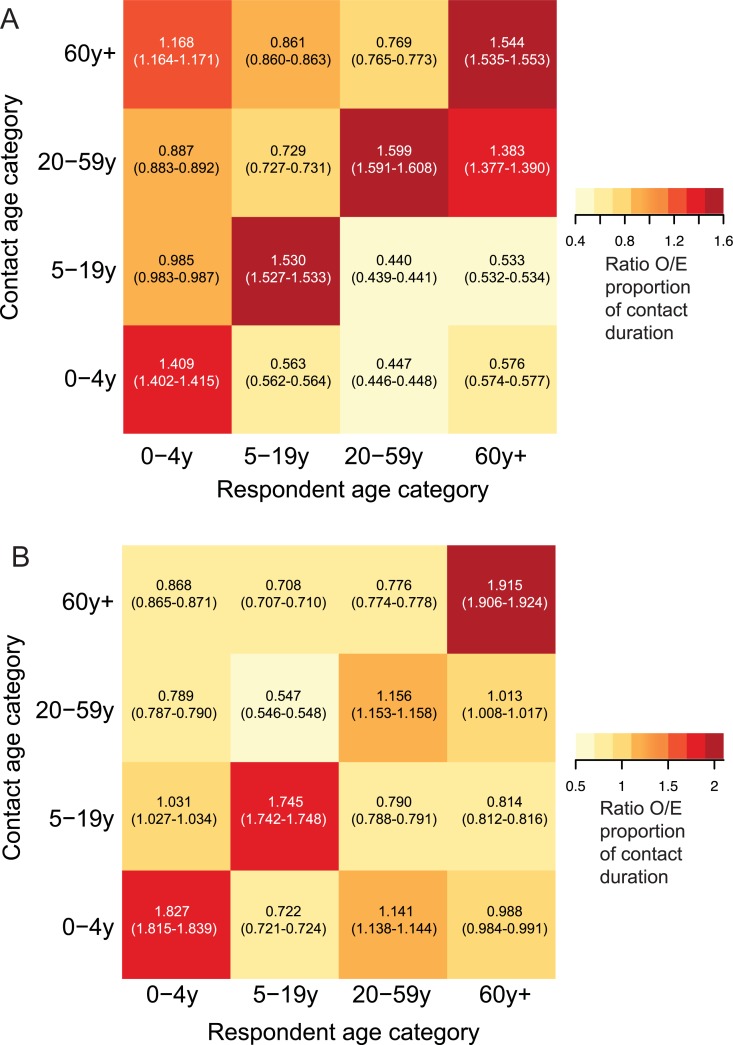
Age-specific mixing matrices. Number of contacts are shown in A, and duration in B. Numeric values represent the ratio of the observed (O) proportion of contacts reported by each respondent age-category with each contact age category to that expected (E) if mixing were proportional to census population proportions in each age category (95% CI).

We quantified the ratio of observed age-specific mixing to a random mixing assumption as described in the methods. As shown in Fig 2A, this ratio is greater than one in all age-assortative categories for number of contacts. The ratio is also greater than one for age-assortative duration of contact. The duration of contact between 20–59 year old adults and 0–4 year old children is greater than would be expected if mixing were proportional, though the number of contacts between these two age categories is not greater than what would be expected if mixing between these age groups were proportional to their relative size in the population (Compare Fig 2A and 2B).

### Factors independently associated with contact number and duration

Adjusted rate ratios are shown in Table 2, with the intercept signifying the average number of contacts or hours in contact for the reference age category (5–19 years age). With number of contacts as our outcome variable and stratifying by gender, we found that respondents from households with 14 people or more had a significantly higher contact rate compared to respondents from the households with 2–6 people after controlling for age and whether contacts were reported for a weekday or weekend (females: adjusted rate ratio 1.37, 95% CI 1.11–1.70; males: adjusted rate ratio 1.30, 95% CI 1.07–1.59). Age was significantly associated with contact rate ratios in both women and men; women of all other age-categories had fewer contacts than 5–19 year old females. Men 20–59 years old had significantly more contacts than 5–19 year old people, whereas male children <5 years and men > = 60 years old had fewer contacts than males 5–19 years old (Table 2). The rate ratio was not different between weekends and weekdays.

**Table 2:** Rate ratios (RRs) for factors influencing the number or duration of human contacts, by gender, Faridibad District, India, October 2015-February 2016.

Characteristic		Number of contacts Adjusted RR (95% CI)*		Duration of contacts Adjusted RR (95% CI)*	
		Males	Females	Males	Females
Age	<1y	**0.44 (0.38–0.51)**	**0.59 (0.52–0.67)**	**0.57 (0.50–0.66)**	**0.65 (0.57–0.73)**
	1-4y	**0.69 (0.59–0.79)**	**0.84 (0.76–0.93)**	**0.84 (0.74–0.96)**	0.96 (0.86–1.07)
	5-19y	Reference	Reference	Reference	Reference
	20-34y	**1.55 (1.26–1.91)**	**0.75 (0.67–0.84)**	**0.74 (0.67–0.81)**	**0.70 (0.64–0.77)**
	35-59y	**1.49 (1.20–1.85)**	**0.83 (0.73–0.95)**	**0.67 (0.60–0.76)**	**0.74 (0.66–0.84)**
	60y+	**0.80 (0.66–0.96)**	**0.83 (0.75–0.93)**	**0.47 (0.41–0.54)**	**0.73 (0.65–0.82)**
Household size	2–6	Reference	Reference	Reference	Reference
	7–9	1.02 (0.83–1.25)	1.08 (0.89–1.31)	1.05 (0.93–1.19)	**1.24 (1.09–1.42)**
	10–13	1.19 (0.96–1.48)	1.13 (0.94–1.36)	**1.15 (1.03–1.29)**	**1.20 (1.07–1.35)**
	14+	**1.30 (1.07–1.59)**	**1.37 (1.11–1.70)**	**1.21 (1.06–1.37)**	**1.38 (1.20–1.58)**
Weekend		1.16 (0.97–1.39)	0.99 (0.89–1.11)	0.95 (0.87–1.04)	1.01 (0.91–1.13)
Intercept†		**22.70 (19.18–26.87)**	**17.83 (15.20–20.90)**	**53.14 (47.98–58.86)**	**40.86 (36.87–45.28)**

Abbreviations: RR, rate ratio; CI, confidence interval. Bold numbers are adjusted rate ratios significant with p-value<0.05.

*Values are exponentiated coefficients from a negative binomial model adjusting for age group, household size group, and weekend/weekday, and accounting for clustering in households.

†Intercept values represent the number or duration of contacts for those in the reference category

Duration spent in contact was significantly higher in households with ten or more people for men, and in households with seven or more people for women. Males 5–19 years old had a longer average contact duration than all other age groups. Females 5–19 years old had a longer average contact duration compared to all other age categories except for 1-4-year-old girls, compared to whom they had similar contact duration (Table 2).

### Contact setting

**Fig 3:**
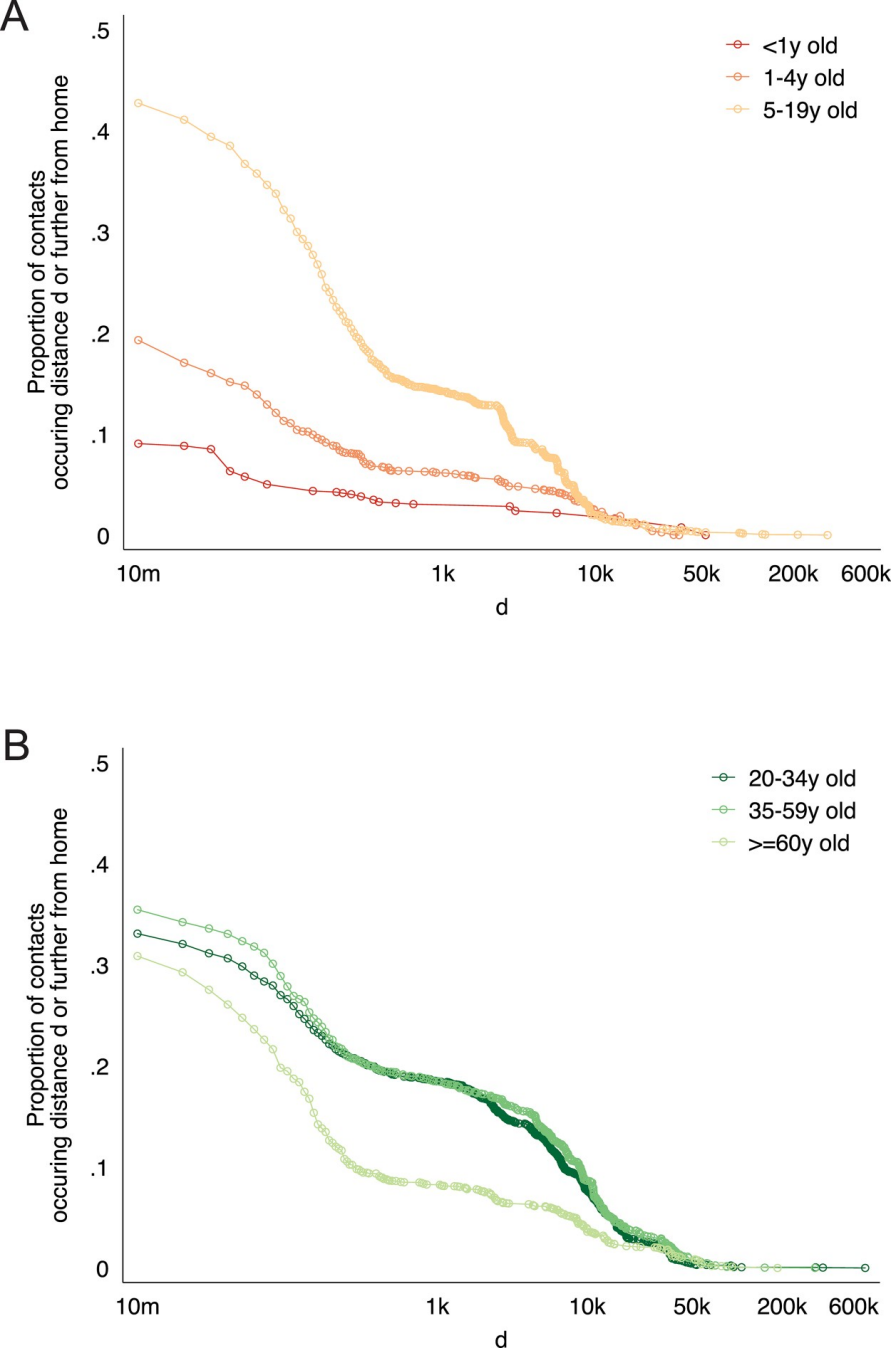
The proportion of contacts reported by respondents in each age-category shown, occurring distance d or further from home.

A very large proportion of contacts overall occurred in the respondent’s home. For infants, 90% of all contacts took place in the home, whereas this number decreased to 80% for 1–4 year old respondents, and to 58% for 5–19 year old respondents. Respondents 5–19 years old also had a larger proportion of their contacts occurring farther from home compared to younger children (Fig 3A). Respondents 20–34 years and 35–59 years old had a larger proportion of their contacts occurring farther from home compared to older adults (Fig 3B).

Of contacts in each setting, 71% of contacts reported at home were reported to involve physical touch, whereas in other settings, the proportions were 85% in transport, 84% in school, 63% at work, and 58% in “other” settings (Fig E in S1 Appendix). Outside the home, we observe contacts between 5-14y old children and 30-39y old adults, most likely school-based contacts between students and teachers. We also observe contacts between older adults, most likely social contacts in the village. Contacts within the home were less age-assortative than were contacts outside the home (Fig F in S1 Appendix).

### Number of contacts by gender

**Fig 4:**
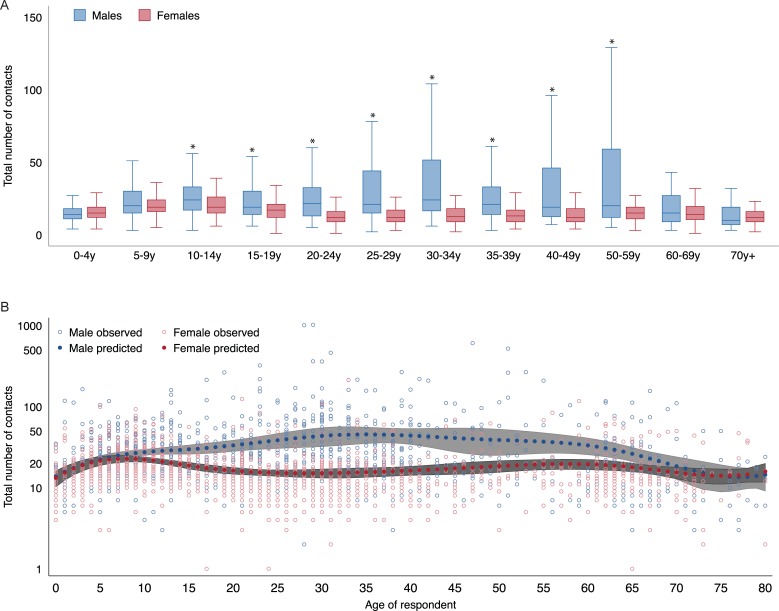
Box plot of total number of contacts by gender and age. Outliers not shown. *signifies a significant difference in the distribution of contact numbers between males and females of the particular age category (A). Observed (open circles) and predicted (filled circles) number of contacts from univariate regression models stratified by gender (male: blue, and female: red), with age splines as predictor variables (B).

Women had fewer total contacts than men in our sample (Kruskal-Wallis χ^2^ = 167.28, dof = 1, p<0.001). Though women had more contacts than did men inside the home (Kruskal-Wallis χ^2^ = 45.04, dof = 1, p<0.001), they made fewer contacts than men outside the home (Kruskal-Wallis χ^2^ = 168.86, dof = 1, p<0.001). The gender disparity in contact rate was not apparent early in childhood and in those > = 60 years old, but appeared in the 5–19, 20–34, and 35-59-year-old age categories. Using a univariate negative binomial regression model with number of contacts as the outcome and age splines as the independent variable, the difference in contacts was apparent starting at age 10 years, and up until age 60 years (Fig 4).

**Fig 5:**
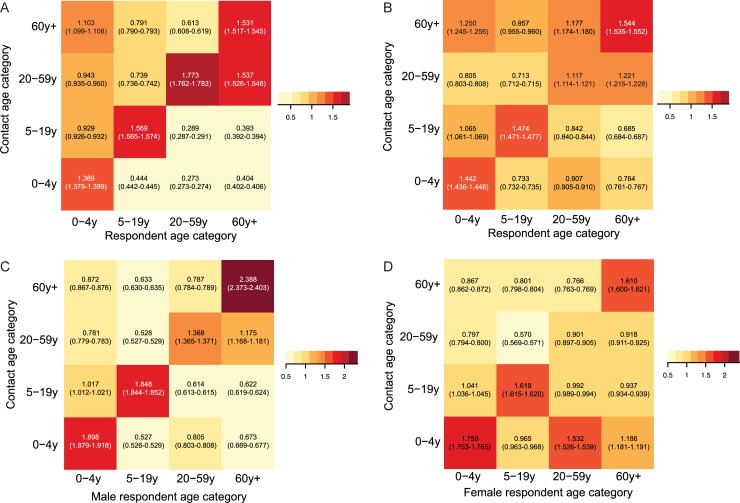
Age-specific mixing matrices for the number (A & B) and duration (C & D) of contacts reported by male (A & C) and female (B & D) respondents. Numeric values represent the ratio of the observed (O) proportion of contacts reported by each respondent age-category with each contact age category to that expected (E) if mixing were proportional to census population proportions in each age category (95% CI).

We also observed that when we stratified respondents by gender, contact number and duration were age-assortative among males as well as females, but adult females 20–59 years and > = 60 years old had a higher than expected contact duration with children 0–4 years old whereas adult males did not (Fig 5).

## Discussion

We found that in a rural area near New Delhi, people reported a median of 17 contacts over the course of a day. Among females, school-age respondents (5–19 years old) made the most contacts whereas among men, working-age respondents made the most contacts. School-age respondents of both genders spent the longest duration in contact with other individuals suggesting that schools could be important places for disease transmission in this rural area. Adult women reported fewer contacts than men, but spent a longer duration in contact with young children than did men. These findings allow us to hypothesize that women may be exposed to respiratory infectious agents at higher rates than adult men due to contact with young children and at the same time, may expose infants to infections due to their close contact with them. These are hypotheses that may be tested using gender- and age-stratified mathematical models of respiratory infection transmission, and that can now be built using the gender-stratified, age-assortative mixing matrices from this study. Findings from our study can be used to inform future models of respiratory infection transmission in India.

### Number, duration, and setting of contacts

We found that the median number of contacts per day among respondents in our rural Indian setting was higher than the median number reported from a study in France [21] that allowed reporting of group contacts (17 vs. 8 contacts per day), similar to the number reported from urban Hong Kong (18 contacts per day) [19], and higher than reported from rural Peru (12 contacts per day) [22]. Our median number of reported contacts was also similar to the average number reported for Italy (20 contacts per day), the European country with the highest average contacts in the POLYMOD study, although the POLYMOD study did not allow reporting of group contacts [18]. Including group contact helps refine previously used methods in contact diaries (capturing all contacts as individuals) by better capturing the right-hand tail of degree distribution through reducing the reporting burden on participants who encountered very large numbers of contacts. Not surprisingly, for our sample characterized by a large household size (median household size = 9), we found that number of contacts and duration increase with household size. As seen in China [20], the duration spent in contact decreases with age compared to school-age children. We found differences in travel patterns between children and adults. Recent studies have suggested that compared to models with age-independent mixing between patches on a grid, models accounting for age-stratified mixing between patches resulted in virus movement rates more consistent with observed rates for multiple influenza strains [39]. Our results of age-specific differences in distances traveled from home may enable models to more accurately capture influenza epidemiology and further the modeling of influenza virus evolution.

### Age-assortativity in contacts

We found the number of contacts to be highly age-assortative, similar to other studies that have reported the greatest degree of assortativity in school-age individuals [40] and young adults [41]. Whereas the duration of contact in our sample was also age-assortative, the 20–59 year old respondents reported spending time with not only their own age-category, but also with 0–4 year old children. This latter pattern reflects parent/grandparent-child mixing.

### Gender differences in reported contacts

Women reported fewer contacts than men in our study. Females have been reported to have *more* contacts than males in the United States [42], and in France [21], but a study in rural Peru found no differences in contacts by gender after controlling for household size and other factors [22]. In South Africa, though no gender differences existed in contacts overall, women had higher rates of contact with children than with men [43]. Women in our study reported a longer duration in contact with children. The interaction between the reduced *number* of contacts, and the increased *duration* of contacts with children warrants further study. Differential exposure to respiratory infection-causing agents by gender (women having a longer duration in contact with young children) might partially explain the higher mortality observed among women in India during the 1918 influenza pandemic [44, 45], and the higher pneumonia mortality among females 5–14 years old [46]. Our study provides the social mixing inputs to build gender- and age-stratified mathematical models of respiratory infection transmission to examine whether differential exposure to agents contributes to gender disparities in mortality in this population.

In addition to providing insights and inputs to future research focused on infectious disease in India, our study highlights social processes of relevance to women’s health and well-being. Starting at age 10 years, females had fewer contacts than males, and this gender-differential pattern in the number of contacts was apparent in all respondents below age 60 years. Our finding of differential contact numbers by gender among adolescent and adult women of childbearing age may reflect the strong male-dominated social structure in this area. In this region, the employment of women outside the home for wages, even on agricultural holdings outside the family, is seen as compromising the family’s standing in the community [47]. Caste, class, gender violence, and a power structure in villages dominated by older, high-caste males have also previously been documented [48]. We may expect the contacts of adolescent girls to be under strict supervision, given the intolerance for female sexuality outside the caste of the family [48]. Our findings highlight the stark difference between girls and boys in potential developmental opportunities that may become available through social contacts.

### Limitations

Contact diaries have several limitations. They suffer from recall bias, [28, 49], and respondents may inaccurately recall the duration of interactions. Additionally, contact diaries are time and resource-intensive means of capturing information, and require time investment on the part of researchers to clarify expectations for respondents [28]. In areas where individual-level (as compared to household-level) penetration of mobile technologies remains limited, contact diaries remain a very useful means of capturing representative interpersonal contact events relevant to the spread of infectious diseases from large numbers of participants [50]. Information derived from contact diaries have also been shown to be informative for transmission models of several infectious diseases [30, 51–53].

In this rural area of India, the validity of self-reported contacts may vary by age and gender, but our findings are supported by the documented bias against women working outside the home and gender power structures in Haryana, India [48]. Furthermore, there existed a trusted relationship between the interviewers—who were part of a team providing free healthcare in the region—and the respondents in our setting, further bolstering confidence in our findings.

Our survey was conducted in a convenience sample of households in five villages. These households were selected due to the presence of children <10 years old and/or the presence of adults > = 60 years old—two demographic sub-groups at high risk of severe outcomes of influenza and other respiratory infections. One should use caution when generalizing these findings; they are most representative of rural areas in Haryana. However, this sampling approach does afford the opportunity to examine contact rates in households that are likely at greatest risk of infection due to the presence of young children and older adults. In addition, contact rates may differ between sick and well respondents. Time series data on contact mixing should be examined along with infection state of individuals in a population to understand how the dynamic nature of contact mixing behavior impacts infection risk, and is impacted by symptomatic infection.

### The field experience

The field experience generated some insights that may be useful for future studies conducting contact surveys. In order to collect location of contact, field workers created a database of locations in the area that people often congregated at. All places of worship and shops in the villages were geocoded and maintained in a database that could be referenced during the survey. In order to survey school children, surveyors had to ensure that they were able to be present at households during the school lunch hour or after the school day. In order to contact people working outside the home, surveyors had to visit the household early or late in the day. Finally, the age at which children were likely to be able to self-report rather than have a caregiver report for them had to be empirically determined. We found that children 5y or younger were generally unable to self-report, and children 6-10y did not answer survey questions in the absence of a caregiver. Best practices for surveying children may differ by location and should be worked out prior to a study.

In conclusion, this study provides estimates of age- and gender-stratified contact rates in rural northern India, and lays the foundation for mathematical and computational models to explore the impact of interventions to reduce ALRI burden.

## Supporting information

S1 Appendix Supplementary figures cited in the text. **Figure A in S1 Appendix. Total number of contacts reported by respondents in each age category**. Pink dots represent mean
**Figure B in S1 Appendix. Total duration (person-hours) in contact reported by respondents in each age category**. Pink dots represent mean
Figure C in S1 Appendix. Proportion of contacts in each duration that were reported to be physical (include touch).
Figure D in S1 Appendix. Proportion of contacts of each frequency that were reported to be physical (include touch).
Figure E in S1 Appendix. Mean percentage of contacts in each location that were physical (included touch).
Figure F in S1 Appendix. Age-assortative mixing matrices for number of contacts in the home (left) and outside home (right).
(PDF)
