# Supplementary material for: Who interacts with whom? Social mixing insights from a rural population in India
Source: PLoS One. 2018 Dec 21;13(12):e0209039. doi: 10.1371/journal.pone.0209039 (PMC6303083; doi:10.1371/journal.pone.0209039)
Supplement: S1 Appendix — Figure A in S1 Appendix. Total number of contacts reported by respondents in each age category. Pink dots represent mean Figure B in S1 Appendix. Total duration (person-hours) in contact reported by respondents in each age category. Pink dots represent mean Figure C in S1 Appendix. Proportion of contacts in each duration that were reported to be physical (include touch). Figure D in S1 Appendix. Proportion of contacts of each frequency that were reported to be physical (include touch). Figure E in S1 Appendix. Mean percentage of contacts in each location that were physical (included touch). Figure F in S1 Appendix. Age-assortative mixing matrices for number of contacts in the home (left) and outside home (right). (PDF) [file pone.0209039.s001.pdf]

Who interacts with whom? Social mixing insights from a rural population in India
Supplementary Material

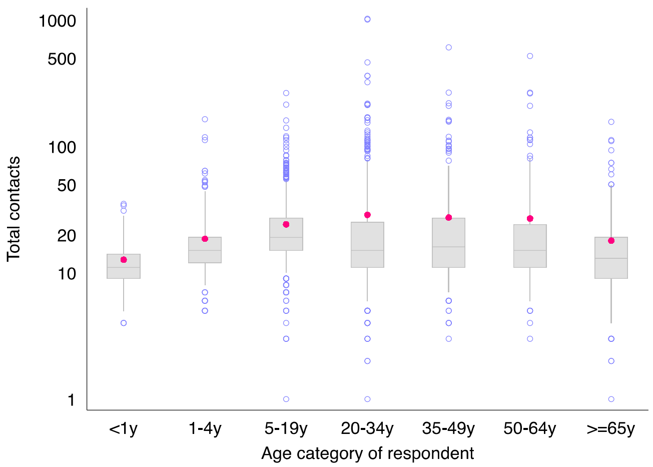

Figure A. Total number of contacts reported by respondents in each age category. Pink dots represent mean.

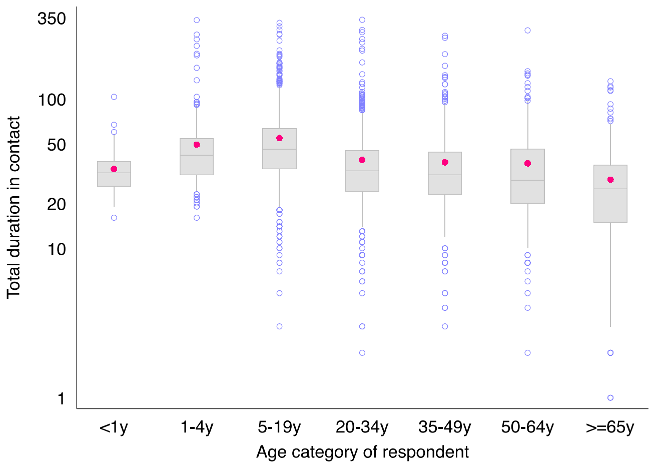

Figure B. Total duration (person-hours) in contact reported by respondents in each age category. Pink dots represent mean.

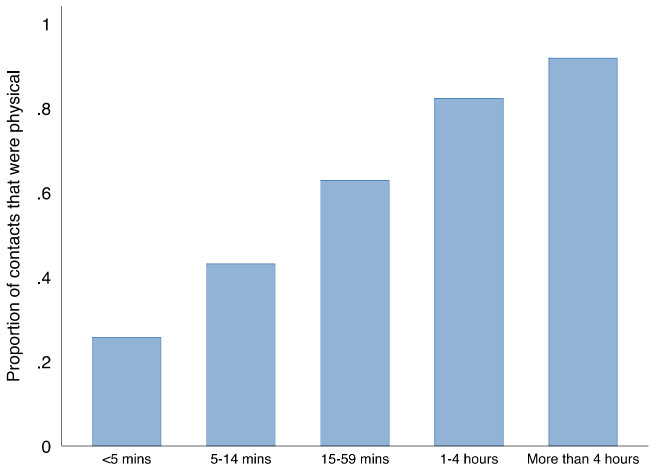

Figure C. Proportion of contacts in each duration that were reported to be physical (include touch).

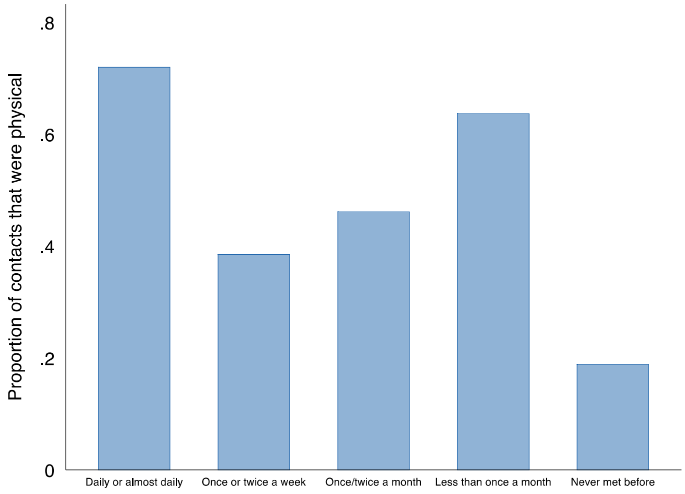

Figure D. Proportion of contacts of each frequency that were reported to be physical (include touch)

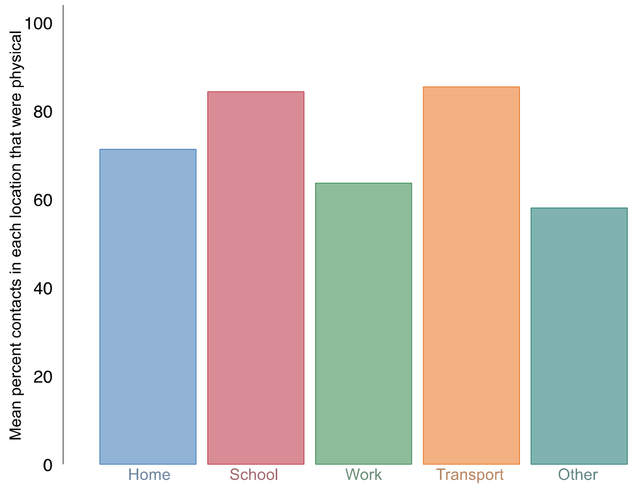

Figure E. Mean percentage of contacts in each location that were physical (included touch).

Who interacts with whom? Social mixing insights from a rural population in India
Supplementary Material

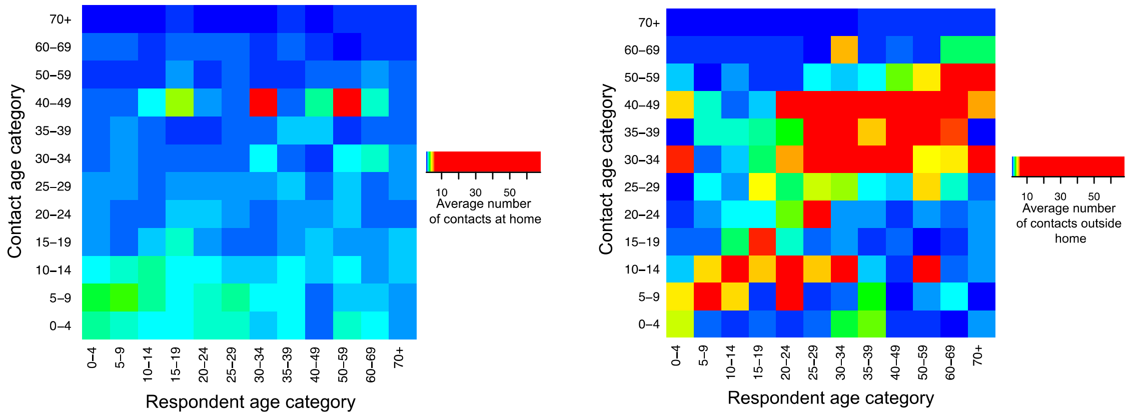

Figure F. Age-assortative mixing matrices for number of contacts in the home (left) and outside home (right).
